# Temperature-Dependent Sex Determination in Fish Revisited: Prevalence, a Single Sex Ratio Response Pattern, and Possible Effects of Climate Change

**DOI:** 10.1371/journal.pone.0002837

**Published:** 2008-07-30

**Authors:** Natalia Ospina-Álvarez, Francesc Piferrer

**Affiliations:** Institut de Ciències del Mar, Consejo Superior de Investigaciones Científicas (CSIC), Barcelona, Spain; Centre National de la Recherche Scientifique, France

## Abstract

**Background:**

In gonochoristic vertebrates, sex determination mechanisms can be classified as genotypic (GSD) or temperature-dependent (TSD). Some cases of TSD in fish have been questioned, but the prevalent view is that TSD is very common in this group of animals, with three different response patterns to temperature.

**Methodology/Principal Findings:**

We analyzed field and laboratory data for the 59 fish species where TSD has been explicitly or implicitly claimed so far. For each species, we compiled data on the presence or absence of sex chromosomes and determined if the sex ratio response was obtained within temperatures that the species experiences in the wild. If so, we studied whether this response was statistically significant. We found evidence that many cases of observed sex ratio shifts in response to temperature reveal thermal alterations of an otherwise predominately GSD mechanism rather than the presence of TSD. We also show that in those fish species that actually have TSD, sex ratio response to increasing temperatures invariably results in highly male-biased sex ratios, and that even small changes of just 1–2°C can significantly alter the sex ratio from 1∶1 (males∶females) up to 3∶1 in both freshwater and marine species.

**Conclusions/Significance:**

We demonstrate that TSD in fish is far less widespread than currently believed, suggesting that TSD is clearly the exception in fish sex determination. Further, species with TSD exhibit only one general sex ratio response pattern to temperature. However, the viability of some fish populations with TSD can be compromised through alterations in their sex ratios as a response to temperature fluctuations of the magnitude predicted by climate change.

## Introduction

Sex determination mechanisms produce the sex ratio, a key demographic parameter crucial for population viability. In gonochoristic vertebrates, sex determining mechanisms can broadly be classified as genotypic (GSD) or temperature-dependent (TSD) [1], [2]. In species with TSD, there are no consistent genetic differences between sexes. The earliest ontogenetic difference between sexes is an environmental one because the ambient temperature during sensitive periods of early development irreversibly determines phenotypic sex and, therefore, the sex ratio [1], [2]. Thus, species with TSD have been proposed to be reliable indicators of the biological impact of global warming, since temperature-induced sex ratio shifts constitute a direct fitness response to thermal fluctuation [3].

So far, predicted effects of climate change on fish populations include distribution shifts [4], alterations in developmental time and larval dispersal [5], decrements in aerobic performance [6], and mismatches in species interactions [7]. Climate change effects on the sex ratio have already been inferred for some sea turtles with TSD [8], [9], but are lacking for fish. Thus, knowledge of the extent to which temperature affects sex ratios is relevant in order to gauge potential threats of rising temperatures on fish populations. Further, knowing the prevalence of TSD is essential for the correct theoretical and empirical study of the evolution of sex determining mechanisms [2], because otherwise inferences on the distribution and prevalence of a particular type of mechanism may be biased [10].

**Figure 1:**
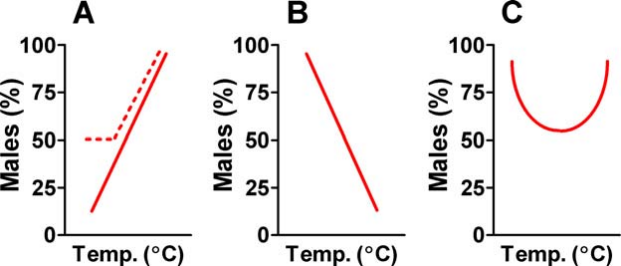
Patterns of temperature-dependent sex determination (TSD) in fish that had been recognized to date. They are defined according to the sex ratio produced as a function of temperature during the thermosensitive period. A, Pattern 1, low temperatures produce female-biased sex ratios and high temperatures produce male-biased sex ratios. B, Pattern 2, low temperatures produce male-biased sex ratios and high temperatures produce female-biased sex ratios. C, Pattern 3, male-biased sex ratios are produced at low and high temperatures, while balanced sex ratios are produced at intermediate temperatures. In some cases, the response may be partial (dashed line in A). The present study demonstrates that fish species with TSD only exhibit pattern 1.

In fish, the first evidence of TSD was obtained in field and laboratory studies carried out in the Atlantic silverside, *Menidia menidia* (F. Atherinopsidae) [11]. Since then, TSD has been claimed in 59 different species (33 of them of the genus *Apistogramma*, F. Cichlidae, and all included in the same study) belonging to 13 families representative of many types of fishes (see Table S1 in the Supplementary Materials). Fish with TSD have readily been grouped according to three patterns of sex ratio response to environmental temperature [12]–[16]: 1, more males at high temperature; 2, more males at low temperature; and 3 more males at extreme (high and low) temperatures (Fig. 1). However, a critical examination of sex ratio produced in response to temperature in fish has never been carried out. Based on all the available data on TSD in fish, it has been reported that 53–55 (including the 33 species of the genus *Apistogramma*), 2–4 and 2 of these species follow patterns 1, 2 and 3, respectively (Table 1). Note that what here are referred to as patterns 1 and 2 of fish essentially corresponds to what in reptiles are referred to as patterns Ib and Ia, respectively. However, pattern 3 of fish is not equivalent to pattern II of reptiles (female-biased sex ratios at low and high temperatures and male-biased sex ratios at intermediate temperatures) but it could be considered an inverse of it.

**Table 1:** Patterns of temperature-dependent sex determination in gonochoristic fish.

		Criteria used here			Confirmation by statistical analyses							
SPECIES	Pattern of TSD previously assigned*	Evidence for the presence of sex chromosomes [Reference]	Sex ratio shift within the RTD (see Suppl. Table 1)	Diagnosis	Lineal regression/F-test							New pattern of TSD proposed here^*^
					n	Intercept	Slope	r^2^	F	DFn/DFd	*P*	
*Carassius auratus*	(1)	Yes [49]	No	GSD+TE	-	-	-	-	-	-	-	0
*Carassius carassius*	1	Yes [15]	No	GSD+TE	-	-	-	-	-	-	-	0
*Danio rerio*	(1)	Yes [38]	No	GSD+TE	-	-	-	-	-	-	-	0
*Gnathopogon caerulescens*	1	Yes [50]	Yes	GSD+TE	-	-	-	-	-	-	-	0
*Misgurnus anguillicaudatus*	1	Yes [51]	Yes	GSD+TE	-	-	-	-	-	-	-	0
*Ictalurus punctatus*	2	Yes [52]	No	GSD+TE	-	-	-	-	-	-	-	0
*Hoplosternum littorale*	1	No	Yes	TSD	16	2.53	2.26	0.40	9.30	1/14	0.009	1
*Oncorhynchus nerka*	2	Yes [53]	No	GSD+TE	-	-	-	-	-	-	-	0
*Menidia menidia*	1	No	Yes	TSD	10	113.79	−995.21	−0.90	70.21	1/8	<0.0001	1
*Menidia peninsulae*	1	No	Yes	TSD	20	−27.81	2.74	0.58	24.61	1/18	0.0001	1
*Odontesthes argentinensis*	1	No	Yes	TSD	9	−55.62	3.91	0.67	14.42	1/7	0.0067	1
*Odontesthes bonariensis*	1	No	Yes	TSD	6	−182.40	9.39	0.98	242.90	1/4	<0.0001	1
*Odontesthes hatcheri*	1	No	No	GSD+TE	-	-	-	-	-	-	-	0
*Oryzias latipes*	(1)	Yes [54]	No	GSD+TE	-	-	-	-	-	-	-	0
*Limia melanogaster*	1	No	Yes	TSD	9	−22.15	2.60	0.69	15.29	1/7	0.0058	1
*Poeciliopsis lucida*	1	No	Yes	TSD	21	−139.50	7.21	0.76	60.91	1/19	<0.0001	1
*Poecilia sphenops*	(1)	Yes [55]	Yes	GSD+TE	-	-	-	-	-	-	-	0
*Sebastes schlegeli*	1	No	No	GSD+TE	-	-	-	-	-	-	-	0
*Dicentrarchus labrax*	1/2	No	No	GSD+TE	-	-	-	-	-	-	-	0
*Apistogramma spp. (33 spp.)* ^†^	1×33	No	Yes	TSD	93	−75.93	4.78	0.75	283.60	1/91	<0.0001	1×33
*Oreochromis aureus*	1	Yes [56]	Yes	GSD+TE	-	-	-	-	-	-	-	0
*Oreochromis niloticus*	1/2	Yes [57]	Yes	GSD+TE	-	-	-	-	-	-	-	0
*Oreochromis mosambicus*	1	Yes [58]	Yes	GSD+TE	-	-	-	-	-	-	-	0
*Paralichthys olivaceus*	3	Yes [59]	Yes	GSD+TE	-	-	-	-	-	-	-	0
*Paralichthys lethostigma*	3	No	No	GSD+TE	-	-	-	-	-	-	-	0
*Pseudopleuronectes yokohamae*	1	Yes [60]	No	GSD+TE	-	-	-	-	-	-	-	0
*Verasper moseri*	1	No	No	GSD+TE	-	-	-	-	-	-	-	0

Abbreviations: TSD, temperature-dependent sex determination; GSD+TE, genotypic sex determination plus temperature effects; RTD, range of temperature during development under natural conditions; n, number of sex ratio datapoints (see Table S1 for references); r^2^ is the correlation coefficient of the regression between temperature and sex ratio produced, whereas F, DFn, DFd and *P* indicate the value of the F-test, the degrees of freedom of the numerator and denominator and the significance, respectively, to determine whether the slope differs from zero, thus indicating that there was a significant effect of temperature on sex ratios. Notes: ^*^Patterns of sex ratio response to temperature: 1, more males at high temperatures; 2, more males at low temperatures; 3, more males at low and high temperatures, as previously assigned based on refs. [12]–[16]; (1) indicates that pattern 1 was not explicitly assigned but that it could be deduced from the data (see Table S1); 0, TSD not supported by data, i.e., thermal effects on GSD (GSD+TE). ^†^Average of the 33 species shown in Table S1.

GSD and TSD can be regarded as two discrete processes that give rise to a continuous pattern of sex determination mechanisms [2], or as two ends of a continuum [17]. In any case, the presence of TSD in a given species is not incompatible with the existence of genotype x environment interactions, which are common in fish, including *Menidia* [15], [18], [19]. However, too often assignment of TSD in many fish species has proceeded regardless of evidence such as the presence of sex chromosomes, which is strongly indicative of GSD [1], [2], [15]. Further, the Atlantic silversides (*Menidia menidia* and *M. peninsulae*) are the only fish species in which the existence of TSD has been demonstrated in the wild; in all other species, data were obtained from laboratory experiments [16]. Thus, evidence to support the presence of TSD has been obtained in many cases using temperatures in the laboratory that the species will rarely experience in nature. It has been pointed out that observed sex ratio shifts under these circumstances might be the consequence of thermal effects on GSD (GSD+TE) rather than proof of the presence of TSD [2], [16]. Thus, there is concern regarding the actual prevalence of TSD in fish. In particular, to discern true cases of TSD from GSD+TE [16]. Nevertheless, the existence of TSD in fish is now widely accepted, assumed to be widespread and expected to be found in more species as new studies become available [10], [12].

The objective of this study was to assess the prevalence of TSD in fish by taking the species where this type of sex determining mechanism has been claimed and applying a series of proposed criteria to discern true cases of TSD from cases of GSD+TE. These included checking for the presence of sex chromosomes and determining whether the temperature used to elicit a change in sex ratios was ecologically relevant, i.e., a temperature that the species usually experiences in nature during the thermosensitive period. We found that TSD is far less widespread that currently thought. We also found that species who actually have TSD exhibit only one single response pattern, not three, producing highly male-biased sex ratios in response to even small increases in temperature. Thus, in one hand, by defining the species that actually have TSD, this study contributes to our understanding of the evolution of sex determining mechanisms. On the other hand, it reports previously unaccounted possible effects of global warming on fish sex ratios.

## Materials and Methods

### Species selection

The 59 species analyzed in this study include all those gonochoristic fishes for which TSD has been explicitly or implicitly assumed as reported in published reviews on the subject [12]–[16], as well as in later publications in the primary literature (Table S1). The species are representative of freshwater, estuarine and marine ecosystems. The only hermaphroditic species where TSD has been claimed, the self-fertilizing cyprinodont *Kryptolebias* (*Rivulus*) *marmoratus*, was not included in our study. In this species, there are no females; essentially all individuals develop as hermaphrodites. Exposure to low temperature during early development increases the proportion of gonochoristic males from ∼3 to 72% [20]. Similarly, the Southern brook lamprey, *Ichthyomyzon gagei*, and the eels, including the American eel, *Anguilla rostrata*, were not included because the circumstantial evidence available so far points to growth-dependent sex differentiation [21] rather than to TSD in these species [22], [23].

### Data collection

For each species analyzed, field data, including the range of natural temperature in which the species can live (RNT), the range of temperature during development in the wild (RTD) as well as the lethal temperature (LT), when available, were obtained from ad hoc reviews, e.g., [24], Fishbase [25], or specific sources, as indicated in Table S1. Experimental (mostly laboratory) data were also compiled from the primary literature, as indicated in Table S1.

### Diagnosis of temperature-dependent sex determination (TSD) as opposed to genotypic sex determination plus temperature effects (GSD+TE)

**Figure 2:**
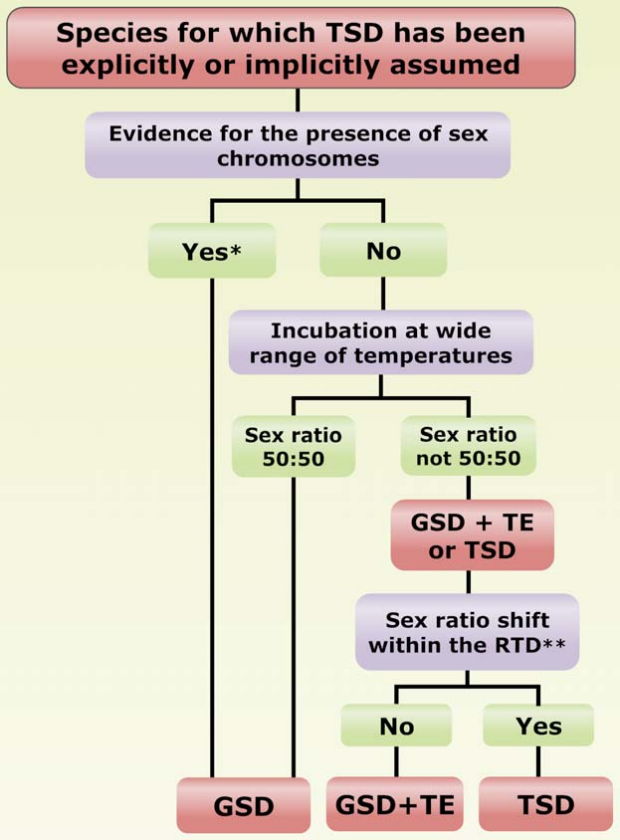
Set of criteria used to determine the presence of temperature-dependent sex determination (TSD) as opposed to genotypic sex determination (GSD), and to distinguish TSD from thermal effects on GSD (GSD+TE). This algorithm is based on the criteria of Valenzuela et al. (2003), and incorporates a modification of the criteria of Conover (2004). See text in the Materials and Methods section for a complete explanation. *Indicates that the evidence for a sex chromosomal system may come from direct (karyotyping, banding) or indirect methods (e.g., progeny analysis of sex-linked traits, mating experiments or crosses with sex-reversed fish). **Indicates that the sex ratio shift must occur within the range of developmental temperatures during development that includes the thermosensitive period (RTD) regardless of whether there is response within the range of natural temperatures where the species lives.

**Figure 3:**
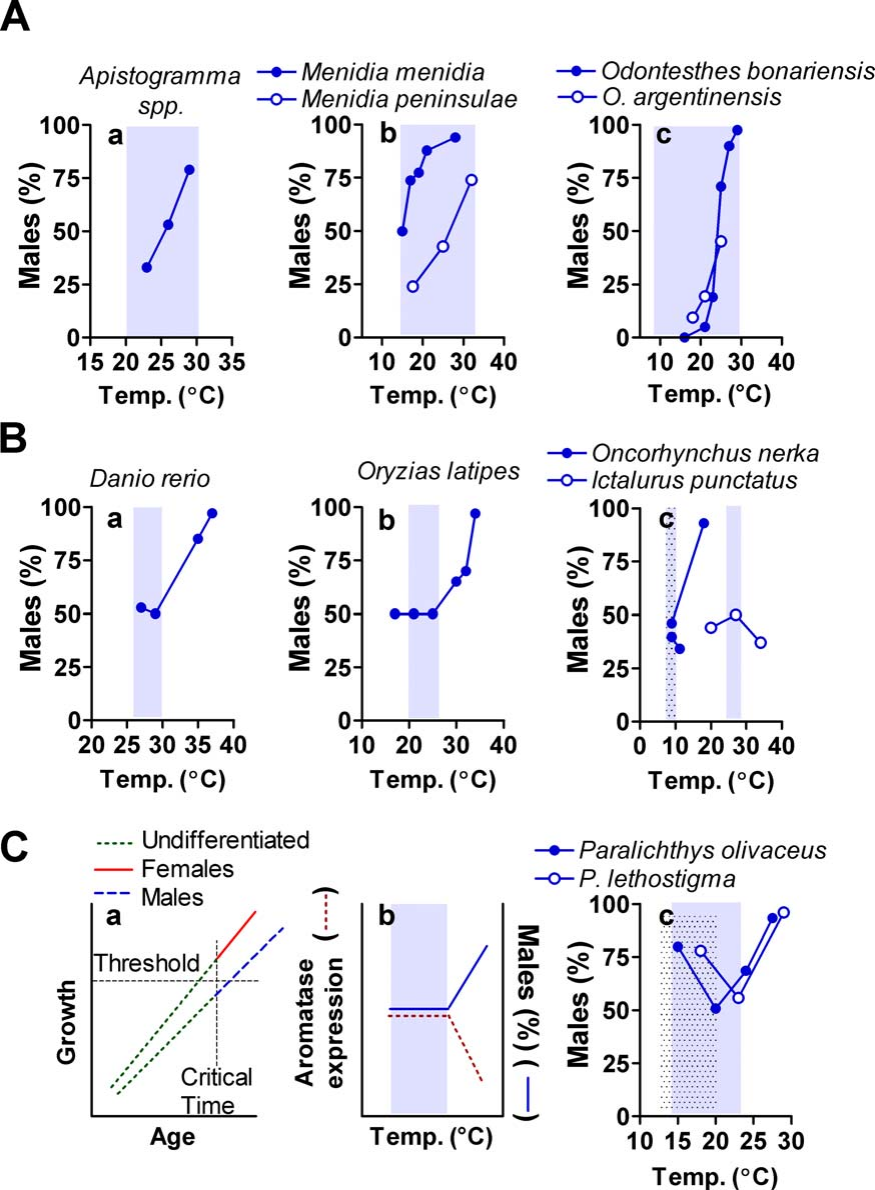
Patterns of sex ratio response to temperature in fish. A, Examples of authentic cases of TSD following pattern 1, more males with increasing temperatures. Sex ratio shifts occur within the range of temperature (shaded areas) normally experienced by fish in the wild. B, Examples of false cases of TSD. Sex ratio shifts only occur at extreme temperatures, and thus represent thermal effects on GSD (a, b). Formerly proposed pattern 2 (c), fewer males at high temperature, is not supported by re-analysis of data (see also Supplementary Table 1). C, Formerly proposed pattern 3, more males at extreme temperatures, can be explained from the combination of two effects unrelated to TSD: slow growing fish at low temperature differentiating as males (a), and the inhibition of aromatase at high temperature causing sex-reversal of genetic females (b). When combined, the two effects result in the observed pattern (c).

To determine the actual prevalence of TSD in fish and to furnish robust patterns of sex ratio response to temperature, we have used a comparative analysis consisting of the application of two independent criteria to identify the presence of TSD (Fig. 2). The first is that of Valenzuela et al. [2], which: (i) stresses that the presence of chromosomal systems of sex determination such as XX/XY or WZ/ZZ, that imply *consistent* genetic differences between sexes, constitutes a very strong evidence of the presence of GSD, and thus it is extremely unlikely that species with these chromosomal systems have TSD. The evidence for sex chromosomes may have been obtained with direct (karyotyping, banding) or indirect methods (e.g., progeny analysis of sex-linked traits, mating experiments or crosses with sex-reversed fish); (ii) considers induced sex ratio shifts that occur only at extreme (but not defined), ecologically irrelevant temperatures, not proof of TSD. The second criteria, which complements the former, is that of Conover [16], which establishes that in order for a species to have TSD, sex ratio shifts in response to temperature fluctuations must occur within a certain range, defined as the range of natural temperature (RNT) in which the species lives. However, since the thermosensitive period in the vast majority of fish examined so far is usually located during early development, and particularly during the larval stages [12]–[16], a modification of the criterion in Conover [16] was used for final assignment of TSD to a given species. Therefore, only those species for which sex ratio shifts occurred not within the RNT but instead within the RTD -the range of temperatures during the period of development that usually includes the thermosensitive period- were considered candidates for having TSD. Particularly in seasonally breeding species of temperate latitudes, RTD is contained within RNT but the opposite is not true (Table S1). Thus, response within the RNT is not enough evidence for TSD. Using the RTD instead of the RNT has the additional advantage of incorporating additional criteria of Valenzuela et al. [2] other than the absence of sex chromosomes, since it facilitates excluding cases of sex reversals induced at extreme temperatures, another possible source of confusion. When a species has a sex chromosomal system and/or sex ratio response to temperature occurring at extreme temperatures (sometimes close to the LT), and definitively outside the RTD (e.g., Fig. 3B), and hence ecologically irrelevant, then TSD is essentially very unlikely. These instances are more appropriately referred to as cases of naturally- or experimentally-induced alterations of genotypic sex determination or genotypic sex determination plus temperature effects (GSD+TE) [2], [16] rather than TSD. Thus, for any given species to have TSD, it should fulfill both of the following two conditions: 1) not having sex chromosomes, and 2) have sex ratio response to temperature within the RTD (Fig. 2). The possible error in proceeding in this manner is negligible and smaller than doing the opposite, i.e., classifying a species as having TSD that has sex chromosomes, which in most cases is strong evidence of GSD, and/or that exhibits sex ratio shifts at artificially high or low temperatures, which is ecologically irrelevant.

### Statistical analysis

Sex ratio deviations from 1∶1 in *Ictalurus punctatus* were checked by applying the Chi-square test [26] to data provided in the original source [27], as depicted in Table S1.

Sex ratio data originally obtained from monosex (all-female) populations exposed to different temperatures were transformed to make them comparable with data obtained with mixed-sex populations of the same species by applying the following formula: Percent males in a 1∶1 (male∶female) population = 50+(percent males in the all-female population/2). Thus, for example, an all-female population that at 20°C the percent of males was 0% and at 28°C was 66% (indicating that two thirds of the females were masculinized) would be equivalent to an 1∶1 population that at 20°C the percent of males was 50% and at 28°C was 50+(66/2) = 83%. Notice that the possibility of producing all-female stocks is indicative that the species in question has a chromosomal system of sex determination, usually of the XX/XY type, thus suggesting the presence of GSD rather than of TSD, as is demonstrated.

The presence of a significant sex ratio response to temperature within the RTD and the verification of the presence of TSD in species diagnosed as having such mechanism of sex determination after applying the criteria explained above was carried out as follows: First, we tested if there was a statistically significant relationship between sex ratio produced and temperature by using the Spearman rank correlation coefficient method. If so, then we compared the slope with the F-test [26] to check whether it was different from zero.

In a few instances, more than one intermediate temperature has been tested. In these cases, for economy of space in the Table S1 only the average sex ratio value, representative for all the intermediate temperatures, is shown. However, for the regressions, all the available intermediate temperatures were used from the original sources. Likewise, each one of the 33 species of the genus *Apistogramma* studied by Römer and Beisenherz [28] was checked individually and the presence of TSD also confirmed statistically on a one-by-one basis, but for simplicity an average result representative of all of them is presented.

In all cases, sex ratio data expressed as percentages (i.e., 100·p, where p is the proportion of males) were arcsin transformed (arcsin of the square root of p) prior to statistical analysis [26]. Statistical analyses and graphs were carried out with the aid of StatGraphics v. 5.1 and Graphpad Prism Software v.4.0.

## Results

**Figure 4:**
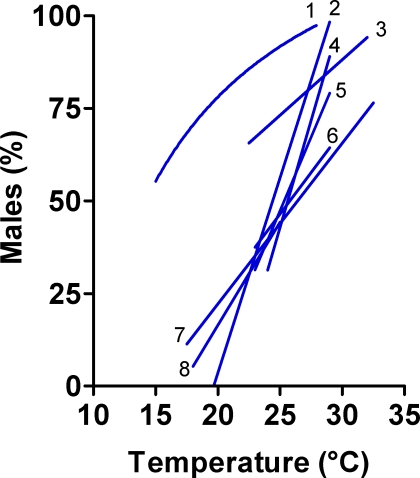
Patterns of sex ratio response to temperature in species of fish with TSD. In all cases, higher temperatures imply a higher number of males produced. Key: 1, *Mendia menidia*; 2, *Odontesthes bonariensis*; 3, *Hoplosternum littorale*; 4, *Poeciliopsis lucida*; 5, average of the 33 *Apistogramma* species; 6, *Limia melanogaster*; 7, *Menidia peninsulae*; 8, *Odontesthes argentinensis*.

Our results show that of the 53–55 species (depending on the authors) previously assigned to pattern 1, the 33 cichlid species of the genus *Apistogramma* indeed exhibit pattern 1 (Fig. 3A a; Table 1) fulfilling the criteria for the assignment of TSD. However, only seven other species of the remaining 20–22 adhere to pattern 1 and have TSD (Fig. 3A b,c). In all but one of the species with TSD the best fit to the experimental data on sex ratio response to temperature was obtained with a linear regression (Y = a+bX). In *Menidia menidia*, however, the best fit was obtained with a reciprocal-X model (Y = a+b/X) (Fig. 4). Included among the species that did not pass the criteria to be diagnosed as true cases of TSD are some established research models such as the zebrafish (*Danio rerio*) and the medaka (*Oryzias latipes*) (Fig. 3B a,b).

Regarding pattern 2, analysis of the original data [27] of channel catfish (*Ictalurus punctatus*) (Table S1) in fact showed no differences with respect to the 1∶1 sex ratio (Chi-square test = 1.42, *P* = 0.233) (Fig. 3B c). Likewise, additional experiments in sockeye salmon (*Oncorhynchus nerka*) reported in Azuma et al. [29] (Fig. 3B c) evidenced the presence of pattern 1 instead of pattern 2, as it had been previously suggested [30]. However, both the channel catfish and the sockeye salmon have sex chromosomes and tested temperatures fall outside the natural range (Table 1). Therefore, these are cases of GSD+TE, not of TSD.

Regarding pattern 3, the two flatfishes previously assigned to this pattern (Fig. 3C), the olive flounder (*Paralichthys olivaceus*) [31] and the Southern flounder (*P. lethostigma*) [32], each failed one of the TSD-determining criteria (Table 1).

Based on the relationship between temperature and sex ratio produced as shown in Table 1, we calculated that fish species with TSD exhibit an average (mean±S.E.M.) pivotal temperature (PT, temperature that produces balanced sex ratios) of 23.3±1.5°C (Table 2). Then, in the scenario of global warming, we took two temperature increases: 1.5 and 4°C, representative of a very likely increase in temperature of water bodies in the upcoming decades and of the maximum predicted increase by the end of this century [33], respectively. With an increase of just 1.5°C, the average number of males in the species with TSD would increase to 61.7±2.1%, and with an increase of 4°C, the average number of males would increase to 78.0±4.1%, i.e., the sex ratios (male∶female) would shift from 1∶1 to ∼2∶1 and to ∼3∶1, respectively (Table 2).

**Table 2:** Pivotal temperature in fish species with TSD and predicted sex ratio shifts with temperature increases.

Species	Pivotal temp. (°C)	Percent of sexes (♂∶♀) at pivotal temp.+1.5°C	Percent of sexes (♂∶♀) at pivotal temp.+4°C
*Apistogramma spp* *	25.3	62 ∶ 38	81 ∶ 19
*Hoplosternum littorale*	18.8	56 ∶ 44	65 ∶ 35
*Limia melanogaster*	25.8	57 ∶ 43	68 ∶ 32
*Menidia menidia*	14.5	61 ∶ 39	75 ∶ 25
*Menidia peninsulae*	26.6	57 ∶ 43	69 ∶ 31
*Odontesthes argentinensis*	25.7	60 ∶ 40	76 ∶ 24
*Odontesthes bonariensis*	24.2	73 ∶ 27	98 ∶ 2
*Poeciliopsis lucida*	25.6	68 ∶ 32	92 ∶ 8
Pivotal temp. (mean±S.E.M.)	23.3±1.5	-	-
Percent males (mean±S.E.M.)	-	61.7±2.1	78.0±4.1

* Average of the 33 species shown in Table S1.

## Discussion

### Prevalence of TSD in fish and response patterns

In reptiles, where TSD was first discovered in vertebrates, this mechanism of sex determination is now well established (see the book by Valenzuela and Lance [34], for reviews). In contrast, in fish, the absolute number of studies is more limited and, significantly, only few of them, concerning the Atlantic silversides, have been carried by samplings in the wild [16], while most have been carried out under controlled laboratory conditions. This may probably reflect the difficulty of sampling fish at different developmental stages in the wild and, especially, correlating environmental variables during critical thermosensitive periods with resulting sex ratios when adults. However, despite these limitations, this situation did not prevent that TSD was until now considered a widespread mechanism of sex determination in fish. Further, based on sex ratio response to temperature, fish species where TSD had been claimed had been grouped into three response patterns.

The analysis of sex ratio response to temperature, considering the scope of such response as well as the presence or not of sex chromosomes, carried out in the present study indicated that many species where TSD had been claimed before are in fact GSD species affected by temperature, i.e., cases of GSD+TE. In GSD+TE species, temperature rather than being the external environmental factor controlling sex determination is capable of affecting the process of gonadal sex differentiation under some circumstances. This distinction is not trivial nor semantic since, according to the canonical definition [1], in TSD species the first ontogenetic difference between sexes is an environmental one (temperature), whereas in GSD+TE species sex determination remains under genotypic control.

Our results support the presence of pattern 1 of sex ratio response to temperature (more males with increasing temperature) but the number of species with TSD is much lower than previously considered and concern mainly species of the families Cichlidae followed by species of the family Atherinopsidae. In addition, we have demonstrated that pattern 2 of sex ratio response to temperature does not exist in fish.

Regarding pattern 3, we propose that this pattern is the result of two independent effects unrelated to TSD (Fig. 3C). First, since exposure to low temperatures decreases growth rates in poikylothermic animals, the increase in males at low temperatures is likely the result of male development according to the threshold model for growth-dependent sex differentiation [21]. Briefly, applied here this model states that when a critical time is reached during development, a sexually undifferentiated gonad will develop as an ovary or as a testis depending on whether it has attained a certain size above or below a threshold, respectively (Fig. 3C a). In fact, a reduction in the number of females was observed among the lower growing fish in the olive flounder, one of the species previously assigned to pattern 3 [35]. Although initial exposure to low temperatures in some cases favors female sex differentiation (as in pattern 1), it is now known that if such exposure is prolonged, thus delaying growth, then male sex differentiation occurs [36]. The preponderance of males at low temperatures also coincides with the left half of pattern 2. Therefore, this pattern sometimes has been also erroneously assigned to species such as the sea bass (*Dicentrarchus labrax*) (Table 1), where growth-dependent sex differentiation occurs [36]. The other effect, the increase in males at high temperatures in species previously assigned to pattern 3, is likely the result of sex-reversal of females as a consequence of the inhibition of aromatase (Fig. 3C b), the enzyme that produces estrogens essential for female sex differentiation in fish [37]. When combined, the two effects produce pattern 3 (Fig. 3C c). In addition, the observed sex ratio response to temperature, especially in the Southern flounder, partly occurs outside the RTD, thus not being representative of true TSD. The inhibition of aromatase at high temperatures –and the consequent increase in the number of males- has also been reported in some species without TSD [15], [38], also explaining why they were assigned to pattern 1, and, interestingly, also seen in many species of reptiles [34], [39]. Thus, we find that only pattern 1 of sex ratio response to temperature is present in fish with TSD (Figure 4), since analysis of the available data does not support the existence of patterns 2 and 3, as accepted until now. This contrasts with the accepted existence of three response patterns in reptiles [34], although perhaps they should be revisited, as done in this study with fish. Further, it has been recognized that the prevalent pattern in reptiles with TSD is pattern Ib [40], which is the equivalent of pattern 1, found to be the only one actually present in fish.

**Figure 5:**
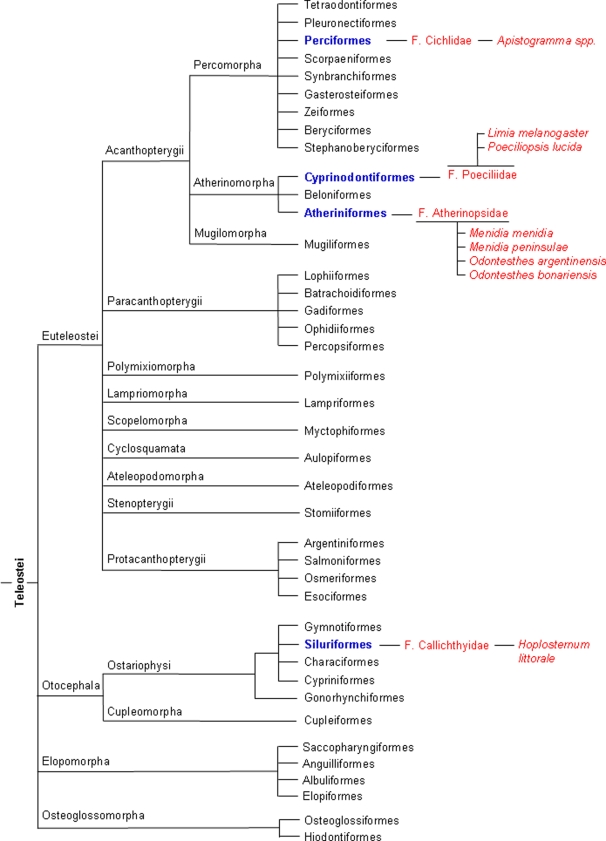
Distribution of temperature-dependent sex determination (TSD) in fish. Orders, families and species with TSD are marked in color. Teleost phylogeny based on Nelson [41].

The results of the present study have implications for our understanding of the evolution of vertebrate sex determining mechanisms. They still agree with the view that TSD has evolved independently many times [1], [2], [40], but we find TSD to be present in only four orders, which include only three of the seven used by Mank et al. [10] to discuss the evolution of sex determining mechanisms specifically in fish. Thus, there is no close relationship among the families where TSD is present (Fig. 5), and many species within the same families are well known for having GSD, suggesting that TSD is clearly the exception in fish sex determination. The phylogenetic distribution suggests that, when it occurs, TSD in fish is a derived rather than an ancestral mechanism. However, there are at least 27,977 known species of teleosts [41] and although admittedly the available data on sex determination are a good representation of the biodiversity, it has to be borne in mind that the number of species examined is still a minority so far. Thus, the picture shown here may change one day as new species are examined in regards to their sex determination mechanisms.

What is the reliability of the original data used to assign TSD in the different species that survived our analysis? In the species of the F. Atherinopsidae (silversides) the evidence seems robust [11], [13], [16], [18], [19], but it should be remembered that the species of the genus *Odontesthes* data has been obtained from laboratory experiments. In the genus *Apistogramma* (South American Cichlids), TSD was demonstrated in many species and thus also seems well established, although the evidence gathered so far originates from a single study [28]. The same situation applies for the atipa, *Hoplosternum littorale*, an Amazonian freshwater fish, where several batches of eggs were used and tested temperatures corresponded to the natural fluctuation; however, data originates from a single study [42]. In contrast, data concerning *Poeciliopsis lucida*, a freshwater fish from Mexico, not only comes from a single study [43] but also the two strains used were highly inbred, one responding to temperature and the other not. The former passed the criteria for being classified as TSD but whether similar results would be obtained with other strains remains to be determined. Further, this is a viviparous species, and viviparity seems incompatible with the requirements to develop TSD [2]. Thus, further research would be necessary to establish whether *P. lucida* has populations with GSD and others with TSD or whether it is a GSD+TE species.

The criteria used here allow the identification of the presence of TSD in a given species. However, this does not exclude the possibility that these species may also have populations with GSD. Therefore, populations with GSD and TSD may co-exist in a single species [16]. Here it is interesting to notice that even in these cases, the pattern of sex ratio response to temperature is invariably pattern 1. On the other hand, it should be noted that the identification of sex chromosomes, particularly if they are homomorphic, can depend on the sensitivity of the method used to search for them. Thus, the number of species with TSD may be further reduced in the future as new technical developments, such as new fluorescent molecular probes, increase our ability to detect sex chromosomes.

The tilapias (genus *Oreochromis*) deserve special attention, not only because their importance for aquaculture but also because some of them constitute established research models where many studies on the effects of temperature in fish sex differentiation have been carried out [12]–[15]. Tilapias did not pass our criteria to be considered TSD species because there are genetic differences between sexes that can be discerned with direct and indirect methods. In fact, currently the genetic sex determinism of tilapias is becoming well understood [44]. Further, recent studies have shown that some tilapia populations adapted to extreme conditions can tolerate temperatures close to 40°C and have rightly pointed out that high temperature influences the normal course of sex differentiation with the resulting masculinization of genetic females [45]. Thus, in accordance with the definitions used here and elsewhere [2], tilapias, then, are a prime example of GSD+TE species, but not of TSD species. To avoid confusion, then, if for a given species there is no compelling evidence of the presence of TSD is better to use the term “temperature effects on sex ratios” or “temperature effects on sex differentiation”, but not “temperature-dependent sex determination”.

### TSD in fish and climate change

How species with TSD will respond to current rapid climate change is a timely question [3], [9], [40]. Some data is available for sea turtles with TSD [8], [9] but is non-existent for fish. Based on the information gathered in the present study some predictions can be made, although it should be taken into account that they are based on a simple linear correlation between temperature and resulting sex ratio. However, in the absence of field data, they are the best educated guess one can make based on the data available so far.

The species identified as having TSD in this study constitute a heterogeneous group since they include both freshwater and marine species living also both in low and high latitudes. Some of them are typically eurithermal while others are stenothermal and, further, they exhibit different reproductive strategies.

Similarly, global warming is not a heterogeneous process, since it affects different parts of the Earth differently. Globally, however, mean temperatures of water bodies are projected to increase by up to ∼4°C by the end of this century according to plausible global change scenarios [33]. Even modest changes of 1–2°C may significantly skew the sex ratio, as already shown in field studies with turtles [3] and sea turtles [9]. In fish, observations made with *M. menidia* eggs collected from the wild have shown that differences of 2°C during the thermosensitive period can result in sex ratio shifts from 50% to 69% males [19].

Thus, the number of females in species with TSD, some of which are of economic or recreational importance, could decrease. One such species is the Argentinean silverside (*O. bonariensis*), where recent studies suggest thermal effects on gonadal development already occurring in natural populations [46]. The species with the least pronounced slopes in the relationship between temperature and the sex ratio produced would be less affected or not affected at all. In *O. bonariensis*, an increase of just 1.5°C could shift the percent males from an average of 50% to ∼73%, that is, from 1∶1 to ∼3∶1. Since the reproductive potential of many fish communities is determined by the number of females available for egg production [47], highly male-biased sex ratios would likely affect population structure and the viability of sensitive stocks.

Potential temperature effects on sex ratios could be difficult to quantify if they are mitigated by other global warming-induced effects, including species distribution shifts [4]. In addition, skewed sex ratios may favor frequency-dependent selection of the less abundant sex, the evolution of TSD towards its disappearance or adjustments in the pivotal temperature [48]. In contrast to past, naturally occurring fluctuations of global temperature, the current climate change event with anthropogenic influences is characterized by its fast pace [33]. Thus, it has been suggested that sensitive species, including species with TSD, could not adapt fast enough to the rapid change in temperatures brought by the new thermal situation [3].

It should be noted that the impact of temperature on sex ratios could also affect species with identifiable sex chromosomes (by causing sex reversal) provided that those effects occur at temperatures within the natural range, or the new shifted range. However, at this point there is insufficient information to determine if, by virtue of their possible higher sensitivity to temperature, species with TSD are better indicators of the impacts of climate change on sex ratios than GSD+TE species.

### Conclusions

In this study, we performed an analysis of field and laboratory data related to fish species for which TSD was assumed. By applying a series of criteria accepted to ascertain the actual presence of TSD, we can reasonably affirm that, excluding the species of the genus *Apistogramma*, in approximately 75% (19 out of 26) of the species considered to have TSD so far, observed sex ratio shifts at extreme temperatures are most likely the consequence of thermal effects on GSD rather than proof of the existence of TSD. Thus, there may be species in which TSD has not yet been discovered but, contrary to the prevailing view, TSD in fish is not as widespread as currently thought, and, importantly, only one general pattern of sex ratio response to temperature exists. However, species which do possess TSD, or species with GSD+TE, may compromise their viability by diminishing the number of females in response to even small increases in water temperatures.

## Supporting Information

Table S1 Temperature-Dependent Sex Determination in Fish. Prevalence, Existence of a Single Sex Ratio Response Pattern, and Possible Effects of Climate Change.
(0.35 MB DOC)
