# Supplementary material for: Temperature-Dependent Sex Determination in Fish Revisited: Prevalence, a Single Sex Ratio Response Pattern, and Possible Effects of Climate Change
Source: PLoS One. 2008 Jul 30;3(7):e2837. doi: 10.1371/journal.pone.0002837 (PMC2481392; doi:10.1371/journal.pone.0002837)
Supplement: Table S1 — Temperature-Dependent Sex Determination in Fish. Prevalence, Existence of a Single Sex Ratio Response Pattern, and Possible Effects of Climate Change. (0.35 MB DOC) [file pone.0002837.s001.doc]

**Supplementary Material**

**Temperature-Dependent Sex Determination in Fish Revisited. Prevalence, a Single Sex Ratio Response Pattern, and Possible Effects of Climate Change**

Natalia Ospina-Álvarez and Francesc Piferrer

Supplementary Table 1 and list of references

**Table S1.** Field and laboratory data used for the assessment of the presence of temperature-dependent sex determination in gonochoristic fish

| ORDER / FAMILY/ SPECIES | Field data | | | | |  | Laboratory data | | | | | | |
| --- | --- | --- | --- | --- | --- | --- | --- | --- | --- | --- | --- | --- | --- |
|  | H | RNT | LT | RTD | References |  | C | % ♂ | I | % ♂ | W | % ♂ | References |
|  | (1) | (2) | (3) | (4) |  |  | (5) |  | (5) |  | (5) |  |  |
| CYPRINIFORMES |  |  |  |  |  |  |  |  |  |  |  |  |  |
| **Cyprinidae** |  |  |  |  |  |  |  |  |  |  |  |  |  |
| *Carassius auratus*^†^ | F* | 0-41* | 39 | 18-20 | 1 |  | 15 | 53 | 23 | 55 | 30 | 92 | 2 ^‡^ |
| *Carassius carassius*^†^ | F* | Tp* | 38-39 | 10-30 | 1, 3 |  | - | - | 24 | 50 | 30 | 61 | 4 |
| *Danio rerio*^†^ | F* | 18-32 | 38-41 | 26-29 | 1 |  | 29 | 50 | 35 | 85 | 37 | 100 | 5 |
| *Gnathopogon caerulescens* | F/E | 4-30 | - | 10-30 | 1 |  | 16 | 64 | 20 | 70 | 30 | 85 | 6 |
| **Cobitidae** |  |  |  |  |  |  |  |  |  |  |  |  |  |
| *Misgurnus anguillicaudatus* | F* | 5-25* | - | 9-23 | 1 |  | 17 | 0 | 20 | 51 | 30 | 90 | 7 ^‡^ |
| SILURIFORMES |  |  |  |  |  |  |  |  |  |  |  |  |  |
| **Ictaluridae** |  |  |  |  |  |  |  |  |  |  |  |  |  |
| *Ictalurus punctatus* | F* | 10-32* | 36 | 25-29 | 1, 3, 8 |  | 20 | 44 | 27 | 50 | 34 | 37 | 9 |
| **Callichthyidae** |  |  |  |  |  |  |  |  |  |  |  |  |  |
| *Hoplosternum littorale* | F/E | 18-33 | 36 | 24-33 | 1, 10 |  | 24 | 46 | 26 | 80 | 30 | 97 | 11 |
| SALMONIFORMES |  |  |  |  |  |  |  |  |  |  |  |  |  |
| **Salmonidae** |  |  |  |  |  |  |  |  |  |  |  |  |  |
| *Oncorhynchus nerka*^†^ | A | 0-25* | 25 | 8-10 | 1, 12 |  | 9 | 40 | 11 | 34 | - | - | 12 |
|  | A | 0-25* | - | 8-10 | 1, 12 |  | 9 | 46 | - | - | 18 | 93 | 13 |
| ATHERINIFORMES |  |  |  |  |  |  |  |  |  |  |  |  |  |
| **Atherinopsidae** |  |  |  |  |  |  |  |  |  |  |  |  |  |
| *Menidia menidia* | M* | 11-33* | 30 -34 | 16-32 | 1, 14 |  | 15 | 50 | 19 | 77 | 28 | 94 | 15 |
| *Menidia peninsulae* | M/E | 10-31 | - | 16-31 | 1, 16 |  | 10-25 | 15 | 20-30 | 43 | - | - | 16 |
|  | M/E | 10-31 | - | 16-31 | 1, 16 |  | 17 | 24 | 25 | 60-70 | 32 | 74 | 17 |
| *Odontesthes argentinensis* | M/E | 8-30 | - | 8-30 | 1, 18 |  | 18 | 10 | 21 | 19 | 25 | 45 | 19 |
| *Odontesthes bonariensis* | F/E | 13-29 | 35-39 | 13-29 | 1, 18, 20 |  | 19 | 0 | 25 | 71 | 29 | 100 | 21 ^‡^ |
| *Odontesthes hatcheri* | F* | 11-24 | - | 16-17 | 1, 19 |  | 13 | 11 | 17-23 | 50 | 25 | 69 | 21 ^‡^ |
| ORDER / FAMILY/ SPECIES | Field data | | | | |  | Laboratory data | | | | | | |
|  | H | RNT | LT | RTD | References |  | C | % ♂ | I | % ♂ | W | % ♂ | References |
|  | (1) | (2) | (3) | (4) |  |  | (5) |  | (5) |  | (5) |  |  |
| BELONIFORMES |  |  |  |  |  |  |  |  |  |  |  |  |  |
| **Adrianichthyidae** |  |  |  |  |  |  |  |  |  |  |  |  |  |
| *Oryzias latipes*^†^ | F* | 5-35 | - | 20-26 | 1 |  | - | - | 27 | 53 | 32 | 67 | 22 |
|  | F* | 5-35 | - | 20-26 | 1 |  | 17 | 50 | 30 | 65 | 34 | 100 | 23 |
| CYPRINODONTIFORMES |  |  |  |  |  |  |  |  |  |  |  |  |  |
| **Poeciliidae** |  |  |  |  |  |  |  |  |  |  |  |  |  |
| *Limia melanogaster* | F* | 22-28* | - | 22-28 | 1 |  | 23 | 39 | 26 | 49 | 29 | 65 | 24 |
| *Poeciliopsis lucida* | F* | 15-35 | - | 15-35 | 1 |  | 24 | 38 | 26 | 54 | 30 | 92 | 25 ^‡^ |
| *Poecilia sphenops* | F/E* | 19-36 | 39-43 | 25-30 | 1, 26 |  | 23 | 11 | 26 | 19 | 30 | 9 | 26 |
| SCORPAENIFORMES |  |  |  |  |  |  |  |  |  |  |  |  |  |
| **Scorpaenidae** |  |  |  |  |  |  |  |  |  |  |  |  |  |
| *Sebastes schlegeli* | M* | Tp* | 32 | 8-17 | 1, 3, 27 |  | 14-17 | 51 | - | - | 27 | 95 | 28 |
| PERCIFORMES |  |  |  |  |  |  |  |  |  |  |  |  |  |
| **Moronidae** |  |  |  |  |  |  |  |  |  |  |  |  |  |
| *Dicentrarchus labrax* | M* | 10-30 | 30-32 | 13-18 | 1 |  | 15 | 100 | - | - | 25 | 85 | 29 |
|  | M* | 10-30 | 30-32 | 13-18 | 1 |  | 13 | 27 | 15 | 31 | 20 | 74 | 30 |
|  | M* | 10-30 | 30-32 | 13-18 | 1 |  | 13 | 89 | - | - | 20 | 68 | 31 |
|  | M* | 10-30 | 30-32 | 13-18 | 1 |  | 13 | 50 | 17 | 57 | 21 | 67 | 32 |
| **Cichlidae** |  |  |  |  |  |  |  |  |  |  |  |  |  |
| *Apistogramma agassizii* | F* | 21-30 | - | 26-29 | 1, 24 |  | 23 | 60 | 26 | 60 | 29 | 82 | 24 |
| *Apistogramma borellii* | F* | 21-30 | - | 24-25 | 1, 24 |  | 23 | 37 | 26 | 64 | 29 | 73 | 24 |
| *Apistogramma cacatuoides* | F* | T* | - | 23-30 | 1, 24 |  | 23 | 20 | 26 | 63 | 29 | 83 | 24 |
| *Apistogramma caetei* | F* | 23-30* | - | 23-30 | 1, 24 |  | 23 | 39 | 26 | 35 | 29 | 31 | 24 |
| *Apistogramma diplotaenia* | F* | 24-29* | - | 24-29 | 1, 24 |  | 23 | 50 | 26 | 53 | 29 | 89 | 24 |
| *Apistogramma eunotus* | F* | 23-30* | - | 23-30 | 1, 24 |  | 23 | 38 | 26 | 53 | 29 | 69 | 24 |
| *Apistogramma geisleri* | F* | 21-29* | - | 23-29 | 1, 24 |  | 23 | 29 | 26 | 51 | 29 | 82 | 24 |
| *Apistogramma gephyra* | F* | 25-30 | - | 23-30 | 1, 24 |  | 23 | 41 | 26 | 54 | 29 | 70 | 24 |
| *Apistogramma gibbiceps* | F* | 27-29* | - | 23-29 | 1, 24 |  | 23 | 19 | 26 | 53 | 29 | 84 | 24 |

| ORDER / FAMILY/ SPECIES | Field data | | | | |  | Laboratory data | | | | | | |
| --- | --- | --- | --- | --- | --- | --- | --- | --- | --- | --- | --- | --- | --- |
|  | H | RNT | LT | RTD | References |  | C | % ♂ | I | % ♂ | W | % ♂ | References |
|  | (1) | (2) | (3) | (4) |  |  | (5) |  | (5) |  | (5) |  |  |
| *Apistogramma gossei* | F*m/e | 23-29* | - | 23-29 | 1, 24 |  | 23 | 29 | 26 | 49 | 29 | 81 | 24 |
| *Apistogramma hippolytae* | F* | 23-30* | - | 23-30 | 1, 24 |  | 23 | 45 | 26 | 49 | 29 | 89 | 24 |
| *Apistogramma hoignei* | F* | 23-29* | - | 23-29 | 1, 24 |  | 23 | 31 | 26 | - | 29 | 82 | 24 |
| *Apistogramma hongsloi* | F* | 21-30 | - | 23-30 | 1, 24 |  | 23 | 29 | 26 | 35 | 29 | 85 | 24 |
| *Apistogramma inconspicua* | F* | 23-28* | - | 23-28 | 1, 24 |  | 23 | 28 | - | - | 29 | 77 | 24 |
| *Apistogramma linkei* | F* | 24-26* | - | 24-26 | 1, 24 |  | 23 | 16 | 26 | 50 | 29 | 89 | 24 |
| *Apistogramma macmasteri* | F* | 23-30* | - | 23-30 | 1, 24 |  | 23 | 30 | 26 | 51 | 29 | 87 | 24 |
| *Apistogramma meinkeni* | F* | 22-29* | - | 22-29 | 1, 24 |  | 23 | 42 | 26 | 54 | 29 | 65 | 24 |
| *Apistogramma nijsseni* | F* | 20-29 | - | 23-29 | 1, 24 |  | 23 | 11 | 26 | 51 | 29 | 85 | 24 |
| *Apistogramma norberti* | F* | T* | - | 23-30 | 1, 24 |  | 23 | 25 | 26 | 52 | 29 | 89 | 24 |
| *Apistogramma ortmanni* | F* | 22-30* | - | 23-30 | 1, 24 |  | 23 | 25 | 26 | 49 | 29 | 83 | 24 |
| *Apistogramma paucisquamis* | F* | T* | - | 23-30 | 1, 24 |  | 23 | 38 | 26 | 54 | 29 | 72 | 24 |
| *Apistogramma pertensis* | F* | 23-30* | - | 23-30 | 1, 24 |  | 23 | 29 | - | - | 29 | 76 | 24 |
| *Apistogramma resticulosa* | F* | T* | - | 23-30 | 1, 24 |  | 23 | 38 | 26 | 39 | 29 | 83 | 24 |
| *Apistogramma staecki* | F* | 24-28* | - | 24-28 | 1, 24 |  | 23 | 28 | 26 | 54 | 29 | 92 | 24 |
| *Apistogramma steindachneri* | F* | 20-25* | - | 20-25 | 1, 24 |  | 23 | 30 | - | - | 29 | 84 | 24 |
| *Apistogramma trifasciata* | F* | 23-30 | - | 23-30 | 1, 24 |  | 23 | 17 | 26 | 49 | 29 | 86 | 24 |
| *Apistogramma uaupesi* | F* | 23-29* | - | 23-29 | 1, 24 |  | 23 | 31 | 26 | 70 | 29 | 91 | 24 |
| *"*Breitbinden" sp. | F* | 23-30 | - | 23-30 | 1, 24 |  | 23 | 26 | 26 | 74 | 29 | 77 | 24 |
| *"*Orangeschwanz" sp. | F* | 23-30 | - | 23-30 | 1, 24 |  | 23 | 34 | 26 | 47 | 29 | 67 | 24 |
| *"*Puerto Narino" sp. | F* | 23-30 | - | 23-30 | 1, 24 |  | 23 | 27 | 26 | - | 29 | 80 | 24 |
| *"*Rio Branco" sp. | F* | 23-30 | - | 23-30 | 1, 24 |  | 23 | 38 | 26 | - | 29 | 67 | 24 |
| *"*Rotpunkt" sp. | F* | 23-30 | - | 23-30 | 1, 24 |  | 23 | 32 | 26 | 43 | 29 | 75 | 24 |
| *"*Samaragd" sp. | F* | 23-30 | - | 23-30 | 1, 24 |  | 23 | 65 | 26 | 71 | 29 | 80 | 24 |
| *Oreochromis aureus* ^†^ | F* | >8* | 41 | 25-35 | 1, 33 |  | 21 | 46 | 27 | 60 | 34 | 98 | 34 |
|  | F* | >8* | 41 | 25-35 | 1, 33 |  | 26 | 97 | 29 | 98 | 32 | 90 | 35 |
|  | F* | >8* | 41 | 25-35 | 1, 33 |  | 27 | 49 | 31 | 60 | 35 | 100 | 33 ^‡^ |

| ORDER / FAMILY/ SPECIES | Field data | | | | |  | Laboratory data | | | | | | |
| --- | --- | --- | --- | --- | --- | --- | --- | --- | --- | --- | --- | --- | --- |
|  | H | RNT | LT | RTD | References |  | C | % ♂ | I | % ♂ | W | % ♂ | References |
|  | (1) | (2) | (3) | (4) |  |  | (5) |  | (5) |  | (5) |  |  |
| *Oreochromis niloticus*^†^ | F*m/e | 14-33* | 42 | >20-33 | 1, 36^¶^ |  | - | - | 28 | 50 | 36 | 99 | 37 |
|  | F* | 14-33* | 42 | >20-33 | 1, 36^¶^ |  | 26 | 56 | 30 | 60 | 37 | 69 | 38 |
|  | F* | 14-33* | 42 | >20-33 | 1, 36^¶^ |  | - | - | 28 | 50 | 35 | 32 | 39 |
|  | F* | 14-33* | 42 | >20-33 | 1, 36^¶^ |  | 18 | 53 | 28 | 50 | 38 | 60 | 40 ^‡^ |
| *Oreochromis mossambicus* | F* | 17-35* | 42 | 20-30 | 1 |  | 20 | 78 | 26 | 59 | 32 | 58 | 34 |
|  | F* | 17-35* | 42 | 20-30 | 1 |  | 20 | 13-38 | 24 | 50-63 | 32 | 40-85 | 41 ^‡^ |
| PLEURONECTIFORMES |  |  |  |  |  |  |  |  |  |  |  |  |  |
| **Paralichthyidae** |  |  |  |  |  |  |  |  |  |  |  |  |  |
| *Paralichthys olivaceus* | M* | ST* | 26-28 | 13-19 | 1 |  | 15 | 80 | 20 | 51 | 28 | 94 | 42 ^‡^ |
| *Paralichthys lethostigma* | M/E* | 5-35 | 29-30 | 14-24 | 1, 43 |  | 18 | 78 | 23 | 55-57 | 28 | 96 | 44 |
| **Pleuronectidae** |  |  |  |  |  |  |  |  |  |  |  |  |  |
| *Pseudopleuronectes yokohamae* | M* | 3-27 | - | 14-17 | 1, 45 |  | 15 | 47 | - | - | 25 | 57-82 | 45 |
| *Verasper moseri* | M* | 4-24 | - | 8-14 | 1, 46 |  | - | - | 14 | 50 | 18 | 100 | 46 |

Abbreviations: (1) H, habitat and/or life cycle: A, anadromous; E, estuarine; F, freshwater; M, marine; (2) RNT, range of natural temperature where the species can live. T, tropical; ST, subtropical; Tp, temperate; (3) LT, lethal temperature; (4) RTD, range of temperature during development under natural conditions; (5) Experimental temperatures: C, cooler; I, intermediate; W, warmer; *Habitats and RNTs marked with one asterisk were obtained through FishBase; ^¶^Some populations are adapted to extreme conditions with temperatures close to 40°C; †Data originally obtained from monosex (all-female) populations were transformed to make them comparable with data obtained with mixed-sex populations. ^‡^Indicates that there was more than one intermediate temperature (see the Statistical Analysis section in the main text). All temperatures are in degrees Celsius. The six Cichlidae species whose names appear within quotation marks (also genus *Apistogramma*) are named as in the original reference [24]. Taxonomy according to Nelson (2006) *Fishes of the World*, 4 ed. (Wiley, New Jersey). Order names appear in uppercase while family names are in boldface.

**References**

1. Froese R, Pauly D (2008) FishBase. Available at: [http://www.fishbase.org](http://www.fishbase.org/)

2. Goto-Kazeto R, Abe Y, Masai K, Yamaha E, Adachi S, et al. (2006) Temperature-dependent sex differentiation in goldfish: Establishing the temperature-sensitive period and effect of constant and fluctuating water temperatures. Aquaculture 254: 617-624.

3. Jobling M (1981) Temperature tolerance and the final preferendum - rapid methods for the assessment of optimum growth temperatures. J Fish Biol 19: 439-455.

4. Fujioka Y (2000) Effects of hormone treatments and temperature on sex-reversal of Nigorobuna *Carassius carassius grandoculis*. Fish Sci 68: 889-893.

5. Uchida D, Yamashita M, Kitano T, Iguchi T (2004) An aromatase inhibitor or high water temperature induce oocyte apoptosis and depletion of P450 aromatase activity in the gonads of genetic female zebrafish during sex-reversal. Comp Biochem Physiol A 137: 11-20.

6. Fujioka Y (2006) Patterns of sex ratio response to water temperature during sex determination in honmoroko *Gnathopogon caerulescens*. Fish Sci 72: 1034-1041.

7. Nomura T, Arai K, Hayashi T, Suzuki R (1998) Effect of temperature on sex ratios of normal and gynogenetic diploid loach. Fish Sci 64: 753-758.

8. Hoar WS, Randall DJ (1988) Fish Physiology, Part A: The Physiology of developing fish: eggs and larvae. New York: Academic Press. 546 p.

9. Patino R, Davis KB, Schoore JE, Uguz C, Strüssmann CA, et al. (1996) Sex differentiation of channel catfish gonads: Normal development and effects of temperature. J Exp Zool 276: 209-218.

10. Hostache G, Pascal M, Kernen M, Tessier C (1992) Température et incubation chez l'atipa, *Hoplosternum littorale* (Teleostei, Siluriforme). Aquat Living Resour 5: 31-39.

11. Hostache G, Pascal M, Tessier C (1995) Influence de la température d’incubation sur le rapport mâle:femelle chez l’atipa, *Hoplosternum littorale* Hancock (1828). Can J Zool 73: 1239-1246.

12. Craig JK, Foote CJ, Wood CC (1996) Evidence for temperature-dependent sex determination in sockeye salmon (*Oncorhynchus nerka*). Can J Fish Aquat Sci 53: 141-147.

13. Azuma T, Takeda K, Doi T, Muto K, Akutsu M, et al. (2004) The influence of temperature on sex determination in sockeye salmon *Oncorhynchus nerka*. Aquaculture 234: 461-473.

14. Fay CW, Neves RJ, Pardue GB (1983) Species profiles: life histories and environmental requirements of coastal fishes and invertebrates (mid-Atlantic): Atlantic silverside. Washington: U.S. Fish and Wildlife Service Biological Services Program. FWS/OBS 82 (11.10). 15 p.

15. Conover DO, Heins SW (1987) The environmental and genetic components of sex ratio in *Menidia menidia* (Pisces, Atherinidae). Copeia 1987: 732-743.

16. Middaugh DP, Hemmer MJ (1987) Influence of environmental temperature on sex ratios in the tidewater silverside, *Menidia peninsulae* (Pisces: Atherinidae). Copeia 1987: 958-964.

17. Yamahira K, Conover DO (2003) Interpopulation variability in temperature-dependent sex determination of the tidewater silverside *Menidia peninsulae* (Pisces: Atherinidae). Copeia 2003: 155-159.

18. Reartes JL (1995) El Pejerrey (*Odontesthes bonariensis*): Métodos de cría y cultivo masivo. Roma: COPESCAL, Documento Ocasional. No. 9.

19. Strüssmann CA, Calsina-Cota JC, Phonlor G, Higuchi H, Takashima F (1996) Temperature effects on sex differentiation of two South American atherinids, *Odontesthes argentinensis* and *Patagonina hatcheri*. Environ Biol Fish 47: 143-154.

20. Cussac VE, Fernández DA, Gómez SE, López HL (2008) Fishes of southern South America: a story driven by temperature. Environ Biol Fish. DOI 10.1007/s 10695-008-9217-2.

21. Strüssmann CA, Saito T, Usui M, Yamada H, Takashima F (1997) Thermal thresholds and critical period of thermolabile sex determination in two atherinid fishes, *Odontesthes bonariensis* and *Patagonina hatcheri*. J Exp Zool 278: 167-177.

22. Sato T, Endo T, Yamahira K, Hamaguchi S, Sakaizumi M (2005) Induction of female-to-male sex reversal by high temperature treatment in medaka, *Oryzias latipes*. Zool Sci 22: 985-988.

23. Hattori RS, Gould RJ, Fujioka T, Saito T, Kurita J, et al. (2007) Temperature-dependent sex determination in Hd-rR medaka *Oryzias latipes*: Gender sensitivity, thermal threshold, critical period, and DMRT1 expression profile. Sex Develop 1: 138-146.

24. Römer U, Beisenherz W (1996) Environmental determination of sex in *Apistogramma* (Cichlidae) and two other freshwater fishes (Teleostei). J Fish Biol 48: 714-725.

25. Sullivan JA, Schultz RJ (1986) Genetic and environmental basis of variable sex-ratios in laboratory strains of *Poeciliopsis lucida*. Evolution 40: 152-158.

26. Hernandez M, Buckle LF, Espina S (2002) Temperature preference and acclimation in *Poecilia sphenops* (Pisces, Poeciliidae). Aquacult Res 33: 933-940.

27. Yamada J, Kusakari M (1991) Staging and the time course of embryonic development in kurosoi, *Sebastes schlegeli*. Environ Biol Fish 30: 103–110.

28. Lee CH, Na OS, Yeo IK, Baek HJ, Lee YD (2000) Effects of sex steroid hormones and high temperature on sex differentiation in black rockfish, *Sebastes schlegeli*. Bull Kor Fish Soc 33: 373–377.

29. Blázquez M, Zanuy S, Carrillo M, Piferrer F (1998) Effects of rearing temperature on sex differentiation in the European sea bass (*Dicentrarchus labrax* L.). J Exp Zool 281: 207-216.

30. Pavlidis M, Koumoundouros G, Sterioti A, Somarakis S, Divanach P, et al. (2000) Evidence of temperature-dependent sex determination in the European sea bass (*Dicentrarchus labrax* L.). J Exp Zool 287: 225-232.

31. Saillant E, Fostier A, Haffray P, Menu B, Thimonier J, et al. (2002) Temperature effects and genotype-temperature interactions on sex determination in the European sea bass (*Dicentrarchus labrax* L.). J Exp Zool 292: 494-505.

32. Mylonas CC, Anezaki L, Divanach P, Zanuy S, Piferrer F, et al. (2005) Influence of rearing temperature at two periods during early life on growth and sex differentiation in two Mediterranean strains of European sea bass (*Dicentrarchus labrax* L.). J Fish Biol 67: 652-668.

33. Desprez D, Mélard C (1998) Effect of ambient water temperature on sex determinism in the blue tilapia *Oreochromis aureus*. Aquaculture 162: 79-84.

34. Mair GC, Beardmore JA, Skibinski DOF. (1990) Experimental evidence for environmental sex determination on *Oreochromis* species. In: Hirano R, Hanyu I, editors; Manila, Philippines. Asian Fisheries Society. pp. 555-558.

35. Baras E, Mpo'n'tcha A, Driouch H, Prignon C, Mélard C (2002) Ontogenetic variations of thermal optimum for growth, and its implication on thermolabile sex determination in blue tilapia. J Fish Biol 61: 645-660.

36. Bezault E, Clota F, Derivaz M, Chevassus B, Baroiller JF (2007) Sex determination and temperature induced sex differentiation in three natural populations of Nile tilapia (*Oreochromis niloticus*) adapted to extreme temperature conditions. Aquaculture 272S1: S3-S16.

37. Baroiller JF, Chourrout D, Fostier A, Jalabert B (1995) Temperature and sex-chromosomes govern sex-ratios of the mouthbrooding cichlid fish *Oreochromis niloticus*. J Exp Zool 273: 216-223.

38. Abucay JS, Mair GC, Skibinski DOF, Beardmore JA (1999) Environmental sex determination: the effect of temperature and salinity on sex ratio in *Oreochromis niloticus* L. Aquaculture 173: 219-234.

39. Kwon J, McAndrew B, Penman D (2002) Treatment with an aromatase inhibitor suppresses high-temperature feminization of genetic male (YY) Nile tilapia. J Fish Biol 60: 625-636.

40. Tessema M, Müller-Belecke A, Hörstgen-Schwark G (2006) Effect of rearing temperatures on the sex ratios of *Oreochromis niloticus* populations. Aquaculture 258: 270-277.

41. Wang LH, Tsai CL (2000) Effects of temperature on the deformity and sex differentiation of tilapia, *Oreochromis mossambicus*. J Exp Zool 286: 534-537.

42. Yamamoto E (1999) Studies on sex-manipulation and production of cloned populations in hirame, *Paralichthys olivaceus* (Temminck et Schlegel). Aquaculture 173: 235-246.

43. Watanabe WO, Carroll P, Daniels HV (2000). Recent progress in controlled reproduction of Southern flounder *Paralichthys lethostigma*. In: U.S.-Japan Natural Resources (UJNR) Aquaculture Panel Meeting. UJNR Tech Rep 28: 141-148.

44. Luckenbach JA, Godwin J, Daniels HV, Borski RJ (2003) Gonadal differentiation and effects of temperature on sex determination in southern flounder (*Paralichthys lethostigma*). Aquaculture 216: 315-327.

45. Goto R, Kayaba T, Adachi S, Yamauchi K (2000) Effects of temperature on sex determination in marbled sole *Limanda yokohamae*. Fish Sci 66: 400-402.

46. Goto R, Mori T, Kawamata K, Matsubara T, Mizuno S, et al. (1999) Effects of temperature on gonadal sex determination in barfin flounder *Verasper moseri*. Fish Sci 65: 884-887.
